# Antioxidant Capacity through Electrochemical Methods and Chemical Composition of *Oenocarpus bataua* and *Gustavia macarenensis* from the Ecuadorian Amazon

**DOI:** 10.3390/antiox12020318

**Published:** 2023-01-30

**Authors:** Carlos Méndez-Durazno, Pablo A. Cisneros-Perez, Bryan A. Loja-Ojeda, Raúl Monge-Sevilla, David Romero-Estévez, Lenys Fernández, Patricio J. Espinoza-Montero

**Affiliations:** 1 Escuela de Ciencias Químicas, Pontificia Universidad Católica del Ecuador, Quito 170525, Ecuador; 2 Facultad de Ciencias Químicas, Universidad Central del Ecuador, Av. Universitaria, Quito 170129, Ecuador; 3 School of Chemical Sciences and Engineering, Yachay Tech University, Urcuqui 100650, Ecuador; 4 Facultad de Ciencias de la Vida, Universidad Regional Amazónica IKIAM, Tena 150102, Ecuador; 5 Centro de Estudios Aplicados en Química, Pontificia Universidad Católica del Ecuador, Quito 170525, Ecuador

**Keywords:** antioxidant capacity, cyclic voltammetry, differential pulse voltammetry, antioxidant index 50, *Oenocarpus bataua*, *Gustavia macarenensis*, antioxidant properties

## Abstract

This study evaluated the antioxidant properties and chemical composition of the seeds, pulp and peels of Ungurahua (*Oenocarpus bataua*) and Pasu (*Gustavia macarenensis*)—fruits, native to the Ecuadorian Amazon. The antioxidant capacity was measured by 1,1-diphenyl-2-picrylhydrazyl (DPPH) and cyclic voltammetry (antioxidant index 50 (AI_50_)) assays; differential pulse voltammetry was used to evaluate antioxidant power using the electrochemical index. The total phenolic content, as well as the yellow flavonoid and anthocyanin content, were quantified via spectrophotometry. In addition, the trans-resveratrol and ascorbic acid content were evaluated through high performance liquid chromatography (HPLC). Ultra-performance liquid chromatography-mass spectrometry (UPLC-MS) was used to identify secondary metabolites with possible therapeutic properties. Results showed that the Pasu peel and seed extracts had the highest antioxidant capacity, followed by the Ungurahua peel; these results were consistent for both spectroscopic and electrochemical assays. HPLC and UPLC-MS analysis suggest that *Oenocarpus bataua* and *Gustavia macarenensis* are important sources of beneficial bioactive compounds.

## 1. Introduction

Fruits and vegetables exhibit high chemodiversity and contain different specialized structures that present varied pharmacological activity [1]. For example, secondary metabolites obtained from fruit have exhibited therapeutic activity against inflammatory processes, oxidative stress and metabolic disorders [2,3,4]. Natural bioactive compounds play a key role in health due to their antioxidant capacity, as well as in the regulation of enzyme activity and gene expression in epigenetic changes [5,6,7,8,9].

Fruits contain high levels of polyphenols with heterogeneous chemical structures. These polyphenols are broadly divided into two major types: flavonoids (anthocyanins, flavonols, flavones, flavanones and isoflavones) and non-flavonoids (phenolic acids and stilbenes) [10,11]. Antioxidant capacity has been attributed to the levels of phenolic compounds, as their specific structures act as reducing agents against reactive oxygen species (ROS) [12,13]. ROS are responsible for the biomolecular damage of proteins, DNA, and lipids, and can induce somatic mutations and neoplastic transformations [14].

Stilbene polyphenols are produced by various families of plants, such as *Dipterocarpaceae, Cyperaceae, Gnetaceae, Pinaceae, Leguminosae, Myrtaceae, Moraceae, Fagaceae, Liliaceae* and *Vitaceae* [15]. Resveratrol (RSV) (3,5,4-trihidroxyestilbene) is one of the main stilbenes that naturally occurs in response to biotic (microbial infections, pathogens) and abiotic (UV radiation, mechanical damage) stress conditions [16,17,18,19]. Major RSV dietary sources include grapes, berries, peanuts, and legumes [15]. In humans, RSV activates and increases SIRT 1 protein levels, which regulate metabolism, stress resistance, immune inflammation functions, and epigenetic factors [20,21,22]. In the search for SIRT 1 activators using pharmacophore libraries, RSV appears to be the most potent molecule [23].

Over the last decade, intensive research has been conducted to identify phytochemicals in native Amazon fruits [24,25,26]. These investigations have found that palm fruits, such as *Euterpe oleracea*, better known as the acai berry, exhibit a significantly high antioxidant capacity. *Oenocarpus bataua* (also known as Seje, Patawa, Chapil and, in this study, Ungurahua), is another Amazonian palm fruit that is found in Ecuador, Colombia, Brazil, and Panama. This species is frequently used by local populations to make nutritious energizing drinks [27,28,29]. The Ecuadorian Amazon, in particular, has a great diversity of fruits that have not been studied, despite being consumed by indigenous peoples as part of their normal diets and, in many cases, as medicinal foods [30].

*Gustavia macarenensis* (also known as Alan paso, Inaco, Inak, Passo, Sachu pasu, and in the current study, Pasu) is a fruit native to the western part of the Ecuadorian and Peruvian Amazon basin. When ripe, its fruits have a delicious flavor and it is mostly consumed locally as juice or oil. Indigenous populations use the pulp, bark, and seeds as traditional medicinal remedies [31]. If systemically investigated, these native fruits have the potential to be marketed globally thanks to their possible applications in the food, pharmaceutical and cosmetic industries.

Therefore, it is important to evaluate key metabolites from new sources, such as the aforementioned fruits, to clarify their potential application in nutraceutical products. Thus, the current study examined the antioxidant properties and bioactive compounds present in ethanolic extracts of the seed, pulp, and peel of *O. bataua* and *G. macarenensis.* The antioxidant capacity was determined using 1,1-dipheny l-2-picrylhydrazyl (DPPH) and antioxidant index 50 (AI_50_) assays, and antioxidant power was evaluated using the electrochemical index (EI). The bioactive constituents of the ethanolic extracts were tentatively identified using high performance liquid chromatography HPLC and ultra-performance liquid chromatography coupled with quadrupole time-of-flight mass spectrometry (UPLC-QTOF-MS) methods.

## 2. Materials and Methods

### 2.1. Reagents

This study used the following reagents: DPPH, the Folin–Ciocalteu (FC) reagent, ethanol, metaphosphoric acid, gallic acid, sodium carbonate, potassium hydroxide, and tetrabutylammonium hexafluorophosphate Bu_4_NPF_6_ (of electrochemical grade); chemical HPLC-grade standards (purity ≥ 99%) of RSV and ascorbic acid (AA) were purchased from Sigma-Aldrich (Darmstadt, Alemania). Methanol, acetonitrile, formic acid and acetic acid were also used, all HPLC-grade; while the other reagents were analytical grade. 

### 2.2. Collection of Fruits Samples

Fruits were purchased from a local supplier in the Mercado Sur (South Market) in the city of Tena, Napo province (1°00′05.5″ S 77°48′44.7″ W), during the fruiting season (January–March 2020). A sufficient number of fruits were purchased according to the relevant guidelines. For the analysis of *O. bataua* and *G. macarenensis,* the fruits were peeled and cut to separate the seeds, peels and pulp (minimum of 1 kg fresh weight for each species). The samples were then cooled to −60 °C in an ultra-low freezer (Infrico Med-CareLTFU40S model). The frozen samples were subsequently lyophilized on an SP Scientific-Genevac (BTP-9E LOVE) for 72 h to a constant weight, kept in the ultra-low freezer at −20 °C, and protected from light until analysis.

### 2.3. Extraction Process

The extraction process was carried out according to the procedure described by Stafussa et al. [32]. Briefly, powered samples (2 g) were extracted using ethanol/water (40:60, 20 mL) at room temperature, which were then stirred on a magnetic stirrer (Mtops Ms300hs) at room temperature at 130 rpm for 120 min while protected from light. The samples were then centrifuged at 5000 rpm for 10 min. The extracts were filtered, transferred to amber bottles, and stored under refrigeration until analysis.

### 2.4. Antioxidant Capacity

#### 2.4.1. DPPH Free Radical Scavenging Assay

The DPPH assay was conducted for all of the ethanolic extract samples according to Stafussa et al. [32]. Briefly, 1.75 mL of DPPH (0.06 mM, in 80% ethanol) was dropped into 0.5 mL of hydroalcoholic extract diluted with 2.75 mL of ethanol, and incubated for 30 min in darkness at room temperature. The absorbance was estimated at 517 nm with a Hach UV-visible DR6000 spectrophotometer. To prepare the external standard calibration curve, standard solutions of AA were used. The results were expressed in μmol of ascorbic acid equivalent per 100 g of fruit in dry weight (AAE 100 g^−1^ d.w.).

#### 2.4.2. Superoxide Anion Assay by Acyclic Voltammetry

Cyclic voltammetry (CV) assays were performed with a potentiostat (CH-Instruments), analyses were performed in a 10 mL electrochemical cell with three electrodes: glassy carbon (3 mm in diameter) as the working electrode, a graphite rod as the counter electrode, and a reference electrode of (Ag/AgCl, 3 M KCl). Before and after each measurement was taken, the surface of the working electrode was polished with alumina in decreasing granulometry (0.3, 0.1 and 0.05 µm), rinsed with distilled water, sonicated for 1 min, rinsed with acetone, and dried with dry air. Dimethylformamide (DMF) containing the supporting electrolyte 0.05 M tetrabutylammonium hexafluorophosphate (Bu_4_NPF_6_) was used as the working solution. A superoxide radical anion (O_2_^•−^) was electrogenerated by reducing the dissolved oxygen (previously saturated by bubbling) in dry DMF. When an O_2_^•−^ constant value oxidation peak has been reached, the addition of extracts starts to increase the antioxidant concentration [33].

The extract´s consumption of O_2_^•−^ was evaluated using the change in the anodic current in the voltammograms, using the dimensionless parameter ($(Ip_{a}^{0}-\;Ip_{a}^{s})/Ip_{a}^{0}$) vs. the concentration of fruit extracts, where $Ip_{a}^{0}$ is the anode peak in the absence of a substrate, and $Ip_{a}^{s}$ is the anodic peak in the presence of a substrate. The reactivity of the extracts was evaluated using the antioxidant index AI_50_. AI_50_ is the concentration of substrate needed to decrease the $Ip_{a}^{0}$ of the O_2_^•−^ by 50% [34]. Low AI_50_ values represent a higher antioxidant capacity of the extracts toward superoxide.

#### 2.4.3. Antioxidant Power According to the Electrochemical Index

Differential pulse voltammetry (DPV) was conducted using the same electrochemical assembly as in the previous determination. The analysis was carried out in a 0.1 M buffer acetate electrolytic solution (pH = 5), using a pulse width of 0.005 s, potential window of −1.2 to 1.6 V vs. Ag/AgCl, pulse amplitude of 0.5 s, and a scan rate of 17 mV s^−1^. The EI was calculated with Equation (1).

(1) $$\mathrm{EI}=\frac{I_{\mathrm{pa}1}}{E_{\mathrm{pa}1}}+\frac{I_{\mathrm{pa}2}}{E_{\mathrm{pa}2}}+\frac{I_{\mathrm{pa}3}}{E_{\mathrm{pa}3}}+\ldots +\frac{I_{\mathrm{pa},n}}{E_{\mathrm{pa},n}}$$

According to previous studies, the EI is directly proportional to antioxidant power [35,36].

### 2.5. Phytochemical Analysis

#### 2.5.1. Total Phenolic Content

The total phenolic content (TPC) was analyzed using the FC method according to Stafussa et al. [32]. A 0.1 mL of ethanol extract was dropped into a volumetric flask containing 7.0 mL of water and 0.5 mL of FC reagent, shaken thoroughly, and left for 3 min. Then, 1.5 mL of 20% (*w/v*) Na_2_CO_3_ solution was added, and the solution was vortex mixed and made up to 10 mL with distilled water. The solution absorbance was measured at 765 nm in a UV-DR6000 spectrophotometer (Hach, Loveland, CO, USA). The results were expressed in mg of gallic acid equivalent (GAE) (100 g^−1^ of fruit in d.w.)

#### 2.5.2. Anthocyanin and Yellow Flavonoid Contents

The total content of anthocyanins and yellow flavonoids were carried out according to Francis et al. [37]. The extraction was performed in a 1:50 (m:v) ratio, the lyophilized sample was homogenized with the extracting solution (1.5 N HCl in 85% ethanol), and extracted for 24 h under refrigeration in a dark room. The extracts were filtered (Whatman no. 1 filter paper). Absorbance was measured at 535 nm (anthocyanins) and 374 nm (yellow flavonoids), respectively, in a Hach UV-visible DR6000 spectrophotometer. Anthocyanin and flavonoid contents were calculated using the absorption coefficients 982 and 766 (g/100 mL)^−1^ cm^−1^, respectively.

#### 2.5.3. Analysis of AA and RSV Using HPLC

The evaluation of the AA content was carried out according to Chebrolu et al. [38]. For the extraction, metaphosphoric acid (MPA, 3.0% *w/v*) was used as a stabilizer. The lyophilized fruits were suspended in the extraction solution at 1:10 ratio under vortex stirring for 2 min and sonicated for 2 min. The extracts were then centrifuged at 3000 rpm. The resulting supernatant was filtered using a 0.45 syringe filter. Separation and quantification of the AA was performed with a reversed-phase-HPLC system. The mobile phase was composed of methanol:water (15:85 v:v), with a pH of 2.5 (adjusted with MPA) and was run isocratically at 0.9 mL min^−1^; UV detection was carried out at 280 nm. Analysis was performed with a Hitachi LaChrom Elite^®^ HPLC (Ichige, Hitachinaka, Ibaraki, Japan) system equipped with a TC C-18 column (150 mm × 4.6 mm i.d., 5 µm) from Agilent.

To quantify the RSV, the Sun et al. [39] method was used with slight modifications. The lyophilized samples in a w:v ratio of 1:40 were extracted with ethanolic solution (ethanol:water, at a 70:30 ratio) in amber vials. The extraction process was carried out in an ultrasonic bath for 35 min. The resulting supernatant was filtered using a 0.45 syringe filter. The separation and quantification of the RSV was performed using a mobile phase, consisting of water-acetonitrile-acetic acid (70:29.9:0.1) at a flow rate of 1 mL min^−1^ and detection with UV at 306 nm. Standard solutions of RSV were prepared in the mobile phase as solvent. Limit of detection (LOD) and limit of quantification (LOQ) were calculated according to the standard deviation of the response (σ) and the slope(s) of the linear regression equation using the following equations:

(2) $$LOD=\frac{3.3\;\times \;\sigma }{s}$$

(3) $$LOQ=\frac{10\;\times \;\sigma }{s}$$

The resulting low values for both the LOD and LOQ demonstrated that the methods had an acceptable sensitivity (Table 1).

#### 2.5.4. Identification of RSV by UPLC-QTOF-MS

The screening and identification of RSV was performed with a Waters Acquity I Class UPLC system with the Xevo G2-XS QTof equipped with an ACQUITY UPLC BEH C18 column (Water, Milford, CT, USA) (1.7 µm, 2.1 × 50 mm i.d.). The chromatographic conditions included 0.01% formic acid in water as solvent A, and 100% acetonitrile as solvent B. The established elution gradient was 1% B (1 min), 1 to 20% B (10 min), 20 to 25% B (10 min), 35 to 50% B (5 min), and a column rebalancing flow rate of 0.25 mL min^−1^. The analysis was carried out with an electrospray ionization (ESI) source, within a mass range of m/z 100 to 1200 Da, negative mode with a capillary voltage of 0.5 kV, cone gas flow of 30 L h^−1^, desolvation gas flow of 900 L h^−1^, source temperature of 140 °C, and a desolvation temperature of 450 °C, with a sample cone and source offset of 40 and 80, respectively. MS/MS experiments were carried out in conjunction with collision energy (CE) ramps: low CE off and high CE from 20 to 30 eV. All data were processed using UNIFI TM v1.8 software under the Mass Lynx NT 4.1 (Waters, Milford, USA) operating interface.

#### 2.5.5. Identification of Bioactive Compounds Based on UPLC-QTOF-MS

The bioactive compounds in the ethanolic extracts obtained from the RSV extraction methodology were determined using UPLC-QTOF-MS. The autoinjector was programmed to inject 10 µL onto the ACQUITY UPLC BEH C18 (100 × 2.1 mm i.d.) column with a particle size of 1.7 μm. Analytical separation was performed using gradient elution consisting of mobile phase A (0.1% formic acid in water) and mobile phase B (0.1% formic acid in acetonitrile), with a gradient of: 0–1 min 1% A, 10 min 1–20% A, 10 min 20–25% A, 5 min 25–35% A, 5 min 35–50% A, and 4 min 1% A. Flow was maintained at 0.2 mL min^−1^.

The optimal conditions were as follows: operating mode (negative), desolvation gas flow (900 L h^−1^), cone gas flow (20 L h^−1^), desolvation temperature (450 °C), source (electrospray ionization), source temperature (140 °C), and capillary voltage (0.5 kV). MS/MS experiments were carried out in conjunction with energy ramp collision (EC): low EC from 6 eV and high EC from 20 to 30 eV. UNIFI™ v1.8.0 software was used for the data processing of the mass spectral features.

### 2.6. Statistical Analysis

All assays were performed in triplicate. Results were recorded as the mean ± standard deviation (SD). Pearson’s correlation analysis was used to determine the correlations between the antioxidant capacity assessment methods.

## 3. Results and Discussion

### 3.1. Antioxidant Properties

Antioxidant compounds from fruits and vegetables are able to mitigate free radicals and prevent the development of chronic diseases [36]; thus, it is essential to explore these molecules and their properties. Accordingly, the demand for rapid and easy-to-use detection approaches to estimate antioxidant performance has increased in the last decade [40,41,42]. To contribute to this field of research, this study determined antioxidant capacity using the single electron transfer (SET) method, which was correlated with the AI_50_ values obtained via electrochemical methods.

**Figure 1:**
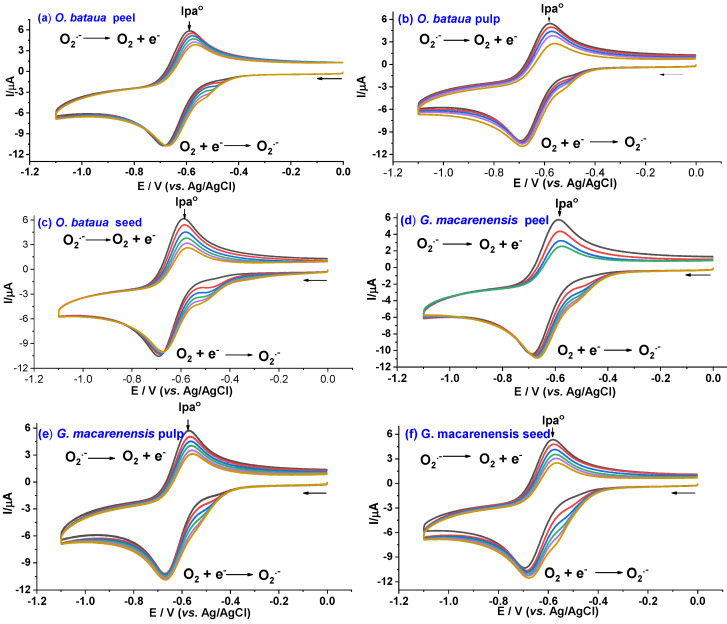
Voltammograms depicting the formation of the radical anion O_2_^•−^ on the surface of a glassy carbon electrode after successively increasing the concentration of the ethanolic extract of (**a**) *O. bataua* peel, (**b**) *O. bataua* pulp, (**c**) *O. bataua* seed, (**d**) *G. macarenensis* peel, (**e**) *G. macarenensis* pulp, and (**f**) *G. macarenensis* seed, in a solution of DMS+ Bu_4_NPF_6_ 0.05 M. Scan rate, 0.1 V/s.

**Figure 2:**
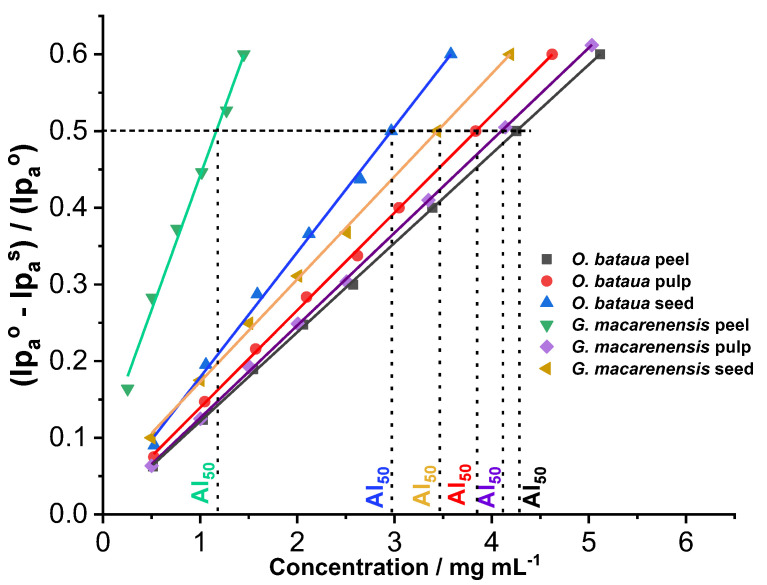
Plots of the dimensionless parameter ($(Ip_{a}^{0}-\;Ip_{a}^{s})/Ip_{a}^{0}$) vs. substrate concentration for *O. bataua* and *G. macaranensis*.

The CV assay is based on the quenching of electrogenerated superoxide; this technique has been used with seaweed, mint, and Ericaceae extracts [43]. In the current study, the CV voltammograms, obtained after adding the substrates to the working solution, showed a decrease in oxidation current (I_pa_) (Figure 1). The dimensionless parameter (($Ip_{a}^{0}-Ip_{a}^{s})/Ip_{a}^{0}$) was determined and plotted as a function of the concentration of the fruit extracts. From the plots, the AI_50_ was obtained (Figure 2); lower values correspond to the extract´s higher antioxidant capacity against the superoxide anion. As shown in Table 2, *G. macarenensis* peel extract had the lowest AI_50_ value; and therefore, the highest antioxidant capacity.

**Figure 3:**
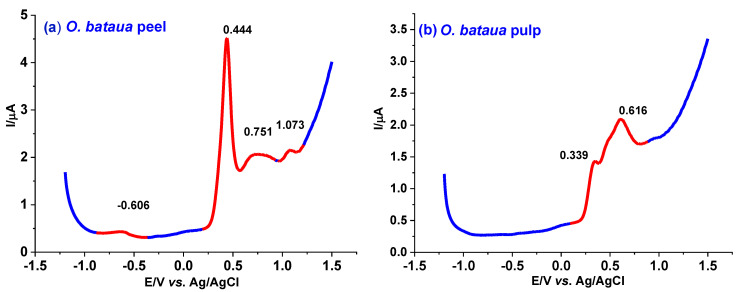

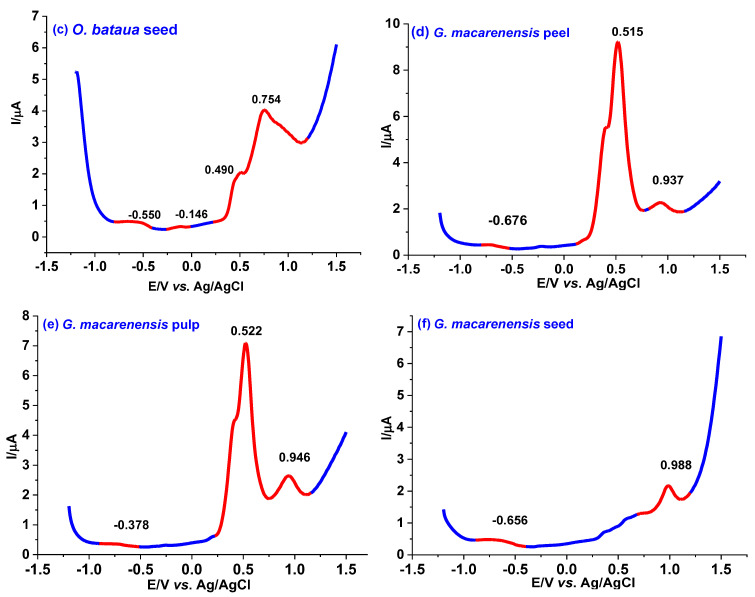
Differential pulse voltammograms for the ethanolic extracts of (**a**) *O. bataua* peel, (**b**) *O. bataua* pulp, (**c**) *O. bataua* seed, (**d**) *G. macarenensis* peel, (**e**) *G. macarenensis* pulp, and (**f**) *G. macarenensis* seed in acetate buffer, pH 4.5, and a scan rate of 17 mV/s.

Antioxidant power was assessed with the EI using DPV. Figure 3 shows the DPV of the fruit extracts. The EI represents the content of nonselective electrochemically oxidized polyphenols [36]. In the voltammograms, the species with high antioxidant capacity that are present in the extracts correspond to low values of oxidation potentials (+0.5 V), and the number of peaks depend on the number of antioxidant species [44]. In Figure 3d, the *G. macarenensis* peel has two anodic peaks around 0.515 V, which correspond to the antioxidant species with high antioxidant capacity. The EI for this extract was 34.57 ± 0.34 μA V^−1^.

The extracts’ antioxidant capacity results, determined by DPPH, AI_50_ and the antioxidant power determined by EI, are shown in Table 2. The peel of *G. macarenensis* had the highest inhibition values of the DPPH radical.

The bioactive compounds of *G. macarenensis* peel extract had the highest antioxidant capacity of all extracts’ DPPH; the AI_50_ and EI values were consistent across all extracts. For instance, the highest antioxidant capacity by DPPH was obtained from *G. macarenensis* peel, which corresponded to the lowest AI_50_ value and the highest EI value. The Pearson´s correlation coefficients of AI_50_, EI, DPPH, and TPC, are given in Table 3. The correlations between the DPPH assay and the electrochemical parameters (AI_50_ and EI) were significant (*p* < 0.05). Therefore, the electrochemical methods can be considered adequate approaches to assess antioxidant capacity in complex matrices, such as fruit. Many studies have shown a positive correlation between antioxidant activities and the TPC of fruit extracts [45,46,47,48,49], which is consistent with the positive correlation between the TPC, DPPH (0.715; *p* ˂ 0.05) and EI (0.635; *p ˂* 0.05) assays found by the current study. Additionally, the AI_50_ was negatively correlated with TPC (*r* = −0.636, *p* < 0.05). As mentioned, the low AI_50_ value corresponds to a high antioxidant capacity, and the negative correlation is due to the decrease in amount of generated superoxide anions as antioxidant capacity increases.

### 3.2. Phytochemical Analysis

Polyphenols are chemical species distributed among a wide variety of fruits and vegetables that have antioxidant and anti-inflammatory biological properties [46]. Further, the phenolic compounds in fruits have been found to have synergistic properties that contribute to their antioxidant capacity [47]. In a previous study, Vasco et al. [48] reported the polyphenol content of 17 fruits from Ecuador; values greater than 500 mg GAE (100 g)^−1^ were classified as high content. The current study’s results for TPC and total anthocyanin and yellow flavonoid content are shown in Table 4. The *G. macarenensis* and *O. bataua* peels (1009.38 ± 1.80 and −1165.87 ± 17.66 mg GAE 100 g^−1^ d.w., respectively) had high TPC values. Data on these fruits’ TPC are scarce in the available literature.

Anthocyanins are natural pigments responsible for the coloration of various fruits and vegetables. The *O. bataua* peel had the highest total content of anthocyanins (46.48 ± 0.31 mg 100 g^−1^ d.w.), followed by *G. macarenensis* pulp (25.57 ± 0.60 mg 100 g^−1^ d.w.), and *O. bataua* pulp (14.82 ± 0.20 mg 100 g^−1^ d.w.). The *G. macarenensis* peel extract (383.59 ± 8.13 mg 100 g^−1^ d.w.) showed a significantly higher flavonoid content compared to other all fruit extracts. No significant difference was observed for yellow flavonoid content in *O. bataua* pulp (55.34 ± 0.29 mg 100 g^−1^ d.w.) or peel (57.17 ± 0.42 mg 100 g^−1^ d. w.) extracts. The yellow flavonoid content in the fruits was consistent with that reported for other tropical fruits [50].

#### 3.2.1. Trans-Resveratrol and Ascorbic Acid Content

Epidemiological studies have shown that RSV acts as an antioxidant and anti-inflammatory agent [51]. RSV was originally extracted by Takaoka et al. [52], in 1939, from the root of *Veratrum grandiflorum O*. Wine grapes (*Vitis vinifera*) and berries contain the highest levels of RSV [53]. The quantification of this compound depends on several factors, including the plant variety, environmental conditions and extraction procedures [54].

**Figure 4:**
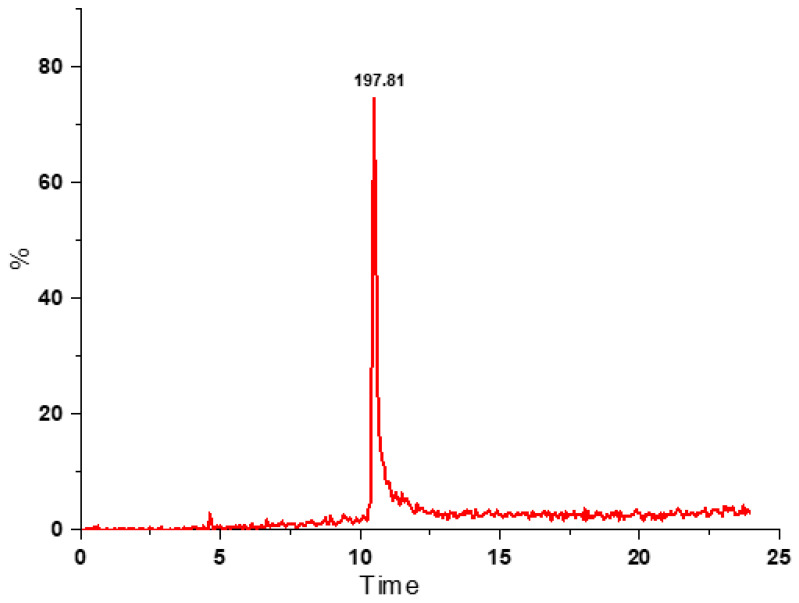
Ultra-performance liquid chromatography-mass spectrometry chromatogram of the extract of *O. bataua* seed.

**Figure 5:**
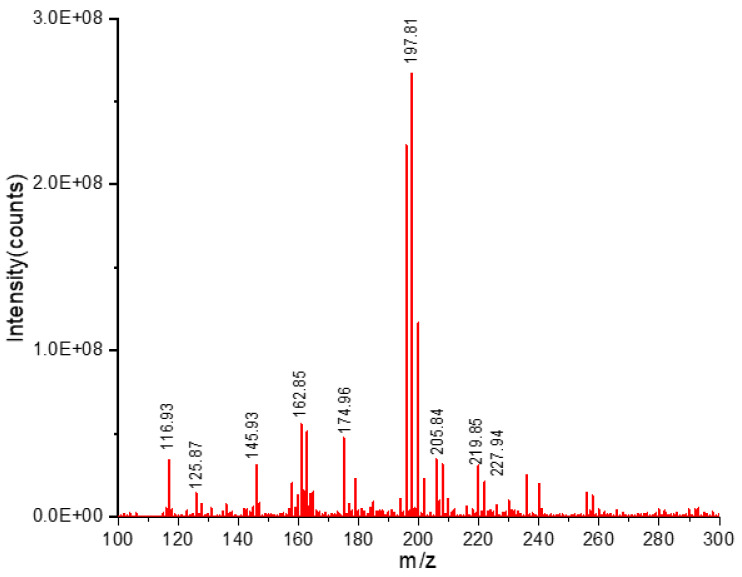
Mass spectrometry spectra of the extract of *O. bataua* seed.

In the current study, RSV was quantified using HPLC, the results are presented in Table 5. RSV content in the *O. bataua* pulp, peel and seed extracts (%R from 90.70 to 98.10%) varied from 1.94 ± 0.10 to 12.33 ± 0.01 µg g^−1^ d.w. RSV content has not previously been reported in exotic fruits from the Ecuadorian Amazon; however, Shrikanta y Col [15] detected a low amount (3.54 μg g^−1^) of RSV in grape seed extracts. Figure 4 shows the chromatogram corresponding to the negative ESI mode of the *O. bataua* seed. The presence of RSV was confirmed via UPLC-QTOF-MS, the select RSV ion was 227 m/z (Figure 5).

According to several studies, citrus fruits are consumed as the main sources of AA, particularly kiwi, cherries and melons, whose AA content exceeds 100 mg/100 g fresh weight [55]. The AA content of the current study is presented in Table 5; it varies from 0.12 ± 0.00 to 15.85 ± 0.06 mg 100 g^−1^ d.b. Among all of the extracts, *G. macarenensis* peel had the highest content of AA (15.85 ± 0.06 mg 100 g^−1^ d.w.). *O. bataua* extracts had the lowest levels of AA. AA levels depend on environmental stress factors (light, temperature, salinity, metals and herbicides), which could explain the differences found [55].

#### 3.2.2. Phenolic Profile and Secondary Metabolites

The ethanolic extracts showed a high diversity of secondary metabolites according to the UPLC-QTOF-MS results. The MS data of the characterized compounds are presented in Table 6; in total, 42 chemical compounds were identified, including phenolic compounds, terpenoids, steroids, alkaloids, coumarin, polyacetylene, peptides and saponin. In both fruits, most phenolic compounds were found in the pulp: 5-O-methyl Shanciguol, Gomisin G, Blestritin C for *O. bataua* and 2,7,2’-trihydroxy-4,4’,7’-trimethoxy-1,1’-biphenantrene and Hordatine A for *G. macarenensis*.

Gomisins are compounds with antioxidant, anticancer and anti-inflammatory properties [56]. Gomisin G has shown therapeutic activity against the human immunodeficiency virus [57]. Furthermore, Yeon et al. [58] reported that Gomisin G may be a potential therapeutic agent for muscle disuse atrophy. Similarly, Hordatines have been found in wheat and barley and possess anti-fungal properties [59]. The coumarins present in *G. macarenensis* could contribute to its yellow color. Finally, in the *G. macarenensis* pulp, the presence of several steroidal saponins/steroidal glycosides were detected, in addition to a peptide.

## 4. Conclusions

The current study is the first to examine the phytochemical components and antioxidant capacity of *O. bataua* and *G. macarenensis* extracts. The electrochemical methods used to evaluate antioxidant capacity showed a significant correlation with the DPPH method. Furthermore, this study also quantitatively detected RSV in *G. macarenensis* extracts. The results support the potential of these less commercialized Amazonian fruit as functional foods, containing bioactive compounds and antioxidants.

## Figures and Tables

**Table 1:** Statistical parameters for the chromatographic analysis of resveratrol and ascorbic acid.

Parameter	Resveratrol	Ascorbic Acid
Limit of detection (μg mL^−1^) Limit of quantification (μg mL^−1^) Linear regression equation Determination coefficient (r^2^) Retention time (min)	0.033 0.089 y = 1.37 × 10^5^ x + 4488.55 0.996 6.1	0.50 1.68 y = 2.63 × 10^5^ x + 4.92 × 10^5^ 0.998 2.46

**Table 2:** Antioxidant properties of *O. bataua* and *G. macarenensis* determined using 1,1-dipheny l-2-picrylhydrazyl (DPPH), antioxidant index 50 (AI_50_), and electrochemical index (EI).

Fruit	Fraction	DPPH AAE (μ*mol AA 100 g^−1^ d.w.*)	AI_50_ (mg mL^−1^)	EI (μA V^−1^)
*O. bataua*	Pulp	478.94 ± 4.85	1.56 ± 0.08	11.92 ± 0.05
	Peel	654.56 ± 0.94	1.47 ± 0.07	15.51 ± 0.16
	Seed	589.44 ± 5.29	1.52 ± 0.10	12.29 ± 0.27
*G. macarenensis*	Pulp	688.40 ± 2.28	1.48 ± 0.02	27.05 ± 0.26
	Peel	1146.41 ± 1.12	0.72 ± 0.04	34.57 ± 0.34
	Seed	523.82 ± 14.09	1.51 ± 0.08	9.06 ± 0.12

**Table 3:** Pearson´s correlation coefficients for AI_50_, EI, DPPH and TPC (total phenolic content).

	AI_50_	EI	DPPH
AI_50_		−0.814	−0.970
EI	−0.814		0.902
DPPH	−0.970	0.901	
TPC	−0.636	0.635	0.715

**Table 4:** Total phenolics content, and anthocyanin and yellow flavonoids content in the two Ecuadorian fruits.

Fruit	Fraction	Total Phenolic Content (*mg GAE 100 g^−1^ d-w*)	Total Anthocyanin Content	Total Yellow Flavonoids Content (mg 100 g^−1^ d.w.)
			(mg 100 g^−1^ d.w.)	
***O. bataua***	Pulp	622.97 ± 4.84	14.82 ± 0.20	55.34 ± 0.29
	Peel	1009.38 ± 1.80	46.48 ± 0.31	57.17 ± 0.42
	Seed	758.25 ± 3.22	14.71 ± 0.02	47.15 ± 0.06
***G. macarenensis***	Pulp	634.30 ± 5.01	25.57 ± 0.60	25.57 ± 0.60
	Peel	1165.87 ± 17.66	9.13 ± 0.11	383.59 ± 8.13
	Seed	153.16 ± 3.45	5.79 ± 0.12	111.39 ± 0.79

**Table 5:** Resveratrol and ascorbic acid content in the evaluated fruit extracts.

Fruit	Fraction	Trans-Resveratrol (µg g^−1^ d.w.)	%Recovery	Ascorbic Acid (mg 100 g^−1^ d.w.)	%Recovery
*O. bataua*	Pulp	1.94 ± 0.10	98.10	0.12 ± 0.00	108.40
	Peel	7.98 ± 0.01	90.90	0.00 ± 0.00	96.40
	Seed	12.33 ± 0.01	90.70	0.00 ± 0.00	118.9
*G.macarenensis*	Pulp	0.00	90.80	5.48 ± 0.24	107.50
	Peel	0.00	95.10	15.85 ± 0.06	96.40
	Seed	< LD	98.10	2.20 ± 0.11	113.40

**Table 6:** Identified compound in *O. bataua* and *G. macarenensis* using UPLC-QTOF-MS analysis.

Fruit	Fraction	Compound Name	Chemical Formula	Experimental m/z Values	t_R_ (min)	Adducts
***O. bataua***	Peel	Pterodontoside E ^a^	**C_21_H_37_O_8_**	417.2495	30.47	-H
	Pulp	5-*O*-Methylshanciguol	**C_30_H_29_O_7_**	501.1905	0.78	+HCOO
		Gomisin G	**C_30_H_31_O_9_**	535.19	1.13	-H
		7-*O*-methylmorroniside ^a^	**C_19_H_29_O_13_**	465.1607	8.67	+HCOO
		Blestritin C	**C_37_H_35_O_8_**	607.2336	8.97	+HCOO
	Seed	Daturametelin H ^b^	**C_23_H_43_O_9_**	463.2905	31.22	+HCOO
		Cireneol G ^c^	**C_17_H_29_O_2_**	265.2181	33.28	-H
***G. macarenensis***	Peel	Oliveramine ^d^	**C_20_H_19_N_2_O_4_**	351.1355	7.08	-H
		Pseudostrychnine ^d^	**C_21_H_22_N_2_O_3_**	395.1624	8.07	+HCOO
		Bisandrographolide C ^a^	**C_40_H_56_O_8_**	709.3923	25.41	+HCOO
	Pulp	1-Acetyl-3-(methoxycarbonyl)-Beta-carboline ^d^	**C_15_H_11_N_2_O_3_**	267.0763	0.65	-H
		Picrasidine U ^d^	**C_31_H_25_N_4_O_7_**	565.1717	0.7	+HCOO
		*Cis*-Osthenone ^e^	**C_14_H_11_O_4_**	243.0666	1.48	-H
		3,4-Dihydro-6,8-dihydroxyl-3-(2’-acetyl-3’-hydroxyl-5’-methoxy phenyl) methyl-1H-[2] benzopyran-1-one ^e^	**C_19_H_17_O_7_**	357.0982	1.57	-H
		Cyclo-(Phe-Tyr) ^d^	**C_18_H_17_N_2_O_3_**	309.1246	3.41	-H
		Qingdainone ^c^	**C_23_H_12_N_3_O_2_**	362.0939	4.27	-H
		2,7,2’-Trihydroxy-4,4’,7’-trimethoxy-1,1’-biphenantrene	**C_31_H_24_O_6_**	491.1489	5.81	-H
		Panaxynol ^f^	**C_17_H_24_O**	243.1751	6.49	-H
		Oliveramine ^d^	**C_20_H_19_N_2_O_4_**	351.1355	7.08	-H
		Denudatine ^d^	**C_22_H_32_NO_2_**	342.2453	8.54	-H
		Bakuchiol ^d^	**C_19_H_25_O_3_**	301.1811	8.96	+HCOO
		Hordatine A	**C_29_H_39_N_8_O_6_**	595.2991	33.22	+HCOO
		Rhodojaponin VI ^a^	**C_21_H_35_O_9_**	431.2273	34.21	+HCOO
	Seed	Bletilol B	**C_28_H_27_O_9_**	507.1642	0.64	+HCOO
		Interiotherin A	**C_30_H_29_O_10_**	549.1768	0.88	+HCOO
		Cyclo-(Phe-Tyr) ^d^	**C_18_H_17_N_2_O_3_**	309.1246	3.41	-H
		Oliveramine ^d^	**C_20_H_19_N_2_O_4_**	351.1355	7.08	-H
		Psamosilenin A ^g^	**C_51_H_64_N_8_O_8_**	961.4797	10.52	+HCOO
		Anemarsaponin G ^h^	**C_50_H_80_O_23_**	1047.4983	18.03	-H
		Kalopanaxsaponin I ^h^	**C_47_H_75_O_18_**	927.494	18.67	+HCOO
		Tenacissoside E ^h^	**C_53_H_78_O_19_**	1017.5051	20.59	-H
		Isoescin IIb ^h^	**C_54_H_84_O_23_**	1145.4379	23.68	+HCOO
		Lablaboside A ^h^	**C_55_H_87_O_25_**	1147.5519	24.14	+HCOO
		Marstenacisside C ^h^	**C_54_H_91_O_26_**	1155.5748	24.15	-H
		Pariphyllin B ^h^	**C_50_H_79_O_21_**	1015.5078	24.33	-H
		Raddeanoside R7 ^h^	**C_53_H_86_O_21_**	1057.5544	26.12	-H
		Raddeanoside R4 ^h^	**C_53_H_85_O_20_**	1041.5665	28.26	-H
		Aesculioside C ^h^	**C_58_H_90_O_24_**	1169.5806	29.42	-H
		Quinquenoside R1 ^h^	**C_51_H_93_O_24_**	1149.6114	30.59	-H
		Ophiopogonin E ^h^	**C_38_H_59_O_13_**	723.3932	32.59	-H
		Borneol-2-O-Beta-D-glucoside ^c^	**C_17_H_29_O_8_**	361.1868	32.59	+HCOO
		Hordatine A	**C_29_H_39_N_8_O_6_**	595.2991	33.22	+HCOO

^a^ Terpenoid. ^b^ Steroid. ^c^ Other compounds. ^d^ Alkaloids. ^e^ Coumarins. ^f^ Polyacetylene. ^g^ Peptide. ^h^ Saponin.

## Data Availability

All the data used for this research are included in this article, there is no additional data elsewhere.
